# P-1252. Individualizing Antibiotic Therapy: Pharmacokinetic Approach to Evaluate and Optimize dosing of Meropenem in Critically Ill Sepsis Patients

**DOI:** 10.1093/ofid/ofae631.1434

**Published:** 2025-01-29

**Authors:** Shakthivel Dhandapani

**Affiliations:** The Tamil Nadu Dr. M.G.R. Medical University, Coimbatore, Tamil Nadu, India

## Abstract

**Background:**

The optimization of antibiotic dosing poses a considerable challenge, particularly in cases of Sepsis with acute renal impairment requiring continuous renal replacement therapy (CRRT). This study centers on the widely prescribed broad-spectrum beta-lactams, specifically meropenem, recognizing its extensive use in severe and complex clinical scenarios. The primary objectives of this study is to formulate new guidelines facilitating dose individualization, thus enhancing therapeutic outcomes for this critical population.

Meropenem dosing recommendations for sepsis shock patients with acute renal impairment

**Figure:**
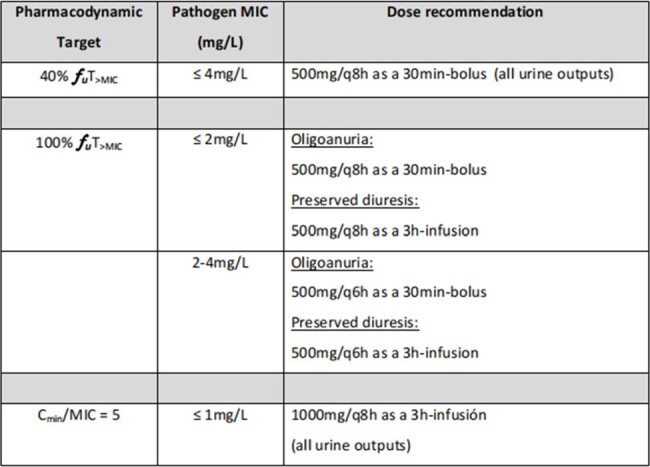

**Methods:**

Three studies were conducted based on specific hypotheses. The initial study involved a comprehensive systematic literature review aimed at critically evaluating the existing evidence regarding the dosing regimens of meropenem. Subsequently, Studies 2 and 3 were undertaken as observational, prospective, open-labeled, multicenter pharmacokinetic investigations within the Intensive Care Unit of a prominent tertiary care hospital in Coimbatore, India. The study cohort comprised thirty patients administered with meropenem experiencing septic shock with acute renal impairment requiring continuous renal replacement therapy. Population Pharmacokinetic models were meticulously developed, validated, and subjected to Monte Carlo simulations for comprehensive analysis.

**Results:**

A comprehensive literature review exposed the limitations of current dosing recommendations, pointing to varied sickness severities, renal function levels, admission diagnostics, CRRT settings, and disparate pharmacokinetic (PK) methodologies. For meropenem, a crucial relationship between 24-hour urine output and total clearance emerged. Oligoanuric patients (< 100mL/24h) displayed a 30% decrease in total clearance compared to those with preserved diuresis ( >500mL/24h). Monte Carlo simulations supported tailored dosing recommendations, addressing bacterial susceptibility and patient diuresis status.

**Conclusion:**

For optimal antibiotic dosing, our findings highlight the importance of considering clinical and demographic factors, including 24-hour urine output and patient weight. These insights can be implemented at the bedside to help navigate the complexities of antibiotic optimization within the Intensive Care Unit.

**Disclosures:**

**All Authors**: No reported disclosures

